# Enhancing dispersion stability of nano zinc oxide with rhamnolipids and evaluating antibacterial activity against harmful corn fungi

**DOI:** 10.3389/fmicb.2025.1527473

**Published:** 2025-06-25

**Authors:** Ben Niu, Shan Qiao, Yiming Sun, Yongwu Niu

**Affiliations:** ^1^National Engineering Research Center For Wheat and Corn Further Processing, Zhengzhou, China; ^2^College of Food Science and Technology, Henan University of Technology, Zhengzhou, China; ^3^Food Laboratory of Zhongyuan, Luohe, Henan, China

**Keywords:** zinc oxide nanoparticles, rhamnolipids, antifungal mechanism, green biosurfactant, particle size, dispersion

## Abstract

**Objective:**

Zinc oxide nanoparticles (ZnONPs) have strong antifungal activity against major harmful fungi in corn kernels. However, due to the high surface energy prone to agglomeration, the residual synthetic surfactants from conventional chemical synthesis may trigger cytotoxicity, whereas rhamnolipids, as a green, safe, non-toxic, and easily degradable biosurfactant, can effectively regulate the size and morphology of zinc oxide nanoparticles, thereby enhancing their antifungal activity and dispersibility.

**Methods and results:**

The products were characterized by one-way experiments with nanoparticle size, zeta potential, ultraviolet-visible spectrum, transmission electron microscopy, Fourier transform infrared spectroscopy and X-ray diffraction to determine the optimization conditions. The results showed that when the concentration of RLs was 1.0 mg/mL, the reaction temperature was 60°C, the concentration of zinc acetate was 0.7 mol/L, and the calcination temperature was 500 °C, the average particle size of RLs-ZnONPs was smaller about 45-50 nm compared with that of the unmodified N-ZnONPs, which had good dispersion and high stability. The antifungal performance of RLs-ZnONPs was evaluated using spore germination rate, mycelial biomass inhibition rate, ergosterol content, and leakage of intracellular contents. It was observed that at a concentration of 4.096 mg/mL, RLs-ZnONPs inhibited the mycelial biomass of four types of fungi by over 76.14%. At the same concentration, spore germination inhibition rates for the same fungi exceeded 86.56%, which interfered with the metabolic activities of the spores and inhibited the germination process. Additionally, RLs-ZnONPs disrupted the stability and integrity of fungal cell membranes, leading to leakage of intracellular electrolytes, nucleic acids, and proteins, thereby suppressing fungal growth.

**Conclusion:**

These research findings indicate that rhamnolipids can significantly improve the dispersibility of nanoscale zinc oxide and effectively reduce its particle size, thereby substantially enhancing its antifungal activity.

## Introduction

At present, food security is seriously threatened by microbial contamination such as fungi (Visconti et al., 2021), which causes huge losses to grain due to fungi and their toxins (Eskola et al., 2020). The effectiveness of physical methods for prevention and control can be impacted by environmental conditions, increased costs, energy consumption, and nutrient destruction (Leitao et al., 1990). Chemical antifungal agents commonly used in chemical methods have high corrosiveness, toxic buildup, poor thermal stability, and biological resistance, limiting their potential applications (Mohapatra et al., 2017). Hence, there is a pressing need to create a safe, environmentally friendly, and effective antifungal agent.

As an antifungal material, zinc oxide nanoparticles (ZnONPs) exhibit a broad antifungal spectrum, strong antimicrobial efficacy, low toxicity, and reduced risk of drug resistance development (Yu et al., 2015). Studies have shown that AgNPs and ZnONPs inhibit the growth of bacterial and fungal strains (Stevanovic et al., 2012; Wahab et al., 2010). At the same time, it can improve the intestinal mucosal morphology, metabolic regulation of nutrients, and enhance immunity of livestock and poultry (Yusof et al., 2021). Studies have demonstrated the antimicrobial efficacy of silver nanoparticles (AgNPs) and ZnONPs against both bacterial and fungal strains (Stevanovic et al., 2012; Wahab et al., 2010). Compared to other nanomaterials, ZnONPs offer cost-effectiveness while enhancing intestinal mucosal integrity, regulating nutrient metabolism, and boosting immunity in livestock and poultry (Yusof et al., 2021). However, the ultrafine particle size of ZnONPs increases their surface energy and tension, rendering them prone to agglomeration. This aggregation tendency directly compromises their antifungal performance (Chen et al., 2022). Furthermore, ZnONPs exhibit structural instability during long-term storage, where particle agglomeration ultimately leads to antimicrobial failure (Huang et al., 2024). Studies have confirmed that the physicochemical properties of ZnONPs are closely correlated with their particle size (Ijaz et al., 2020). Current synthesis methods face two primary limitations: insufficient precision in controlling product morphology and microstructure, and difficulties in achieving cost-effective large-scale production (Jiang et al., 2023).

To address ZnONPs synthesis challenges, researchers have developed novel green preparation methods. Green synthesis of nanomaterials offers advantages over physical and chemical methods, including simplicity, cost-effectiveness, and environmental friendliness. Bio-based stabilizers and reducing agents effectively minimize nanoparticle agglomeration (Rani et al., 2023; Piro et al., 2023). Rahimzadeh et al. employed natural phytochemicals from plant extracts as reducing and capping agents, successfully synthesizing spherical SiO_2_ nanoparticles with excellent dispersion and superior stability compared to traditional chemical methods (Rahimzadeh et al., 2022). Majedi et al. utilized dill leaf extract as a bio-nanocatalyst to synthesize monodisperse spherical zinc oxide nanoparticles with high crystallinity. These nanoparticles demonstrated promising anticancer properties through a simple, eco-friendly, and cost-effective process (Majedi et al., 2023). Azeez et al. prepared spherical ZnONPs with an average size between 30 and 35 nm using celery graveolens L. leaf extract as an efficient chelating and capping agent, and the product removed methyl orange organic pollutant from water within 3 min of UV irradiation (Azeez and Barzinjy, 2020).

In recent years, there has been significant research on the synthesis and preparation of surfactant-modified nanomaterials (Mary and Bose, 2018; Table 1). Among them, rhamnolipids (RLs) are natural biosurfactants secreted by microorganisms such as Pseudomonas aeruginosa, with environmentally friendly and degradable properties and have been found by some scholars to have better surface properties as a biosurfactant than chemical surfactants (Cazals et al., 2022). It is characterized by good surface activity, being environmentally friendly, safe, non-toxic, and easily degradable (Varjani et al., 2021; Chong and Li, 2017). ZnONPs particles with particle size of 40-50 nm were biosynthesized using rhamnolipids, and the experiments showed that ZnONPs modified by rhamnolipids exhibited significantly enhanced antimicrobial and anti-biofilm activities, and the inhibition rates of ZnONPs at 250 μg/mL against the pathogenic bacteria and the biofilm were up to 80 and 78%, respectively (Malakar et al., 2021). The method is centered on biomolecules and significantly reduces the environmental burden, which is a typical green synthesis method (Azeez and Barzinjy, 2020). Additionally, rhamnolipids also possess strong and antifungal properties (Sha et al., 2017). The enhancement of their antifungal activity is expected through the synthesis and modification of nano- antifungal materials.

**TABLE 1:** Related research on nanomaterials.

Types of nanomaterials	Preparation method	Findings	References
CuONPs	CuO nanoparticles were synthesized by hydrothermal method and functionalized/capped with RLs.	The minimum inhibitory concentration (MIC) was 7.8 μg/L for Gram-negative bacteria and 250 μg/L for Gram-positive bacteria, and the MIC values against Candida albicans and Aspergillus niger, which are fungi, were observed at 125 μg/L and 62.5 μg/L, respectively.	(Athira et al., 2021)
CuONPs	RL-terminated CuONPs were synthesized by hydrothermal method using RLs as biosurfactants.	Antimicrobial tests were performed on RL-CuO NPs.RLs capped CuO NPs showed antimicrobial activity at concentrations much lower than those of individual RL, CuO. The developed RL-CuO NPs were incorporated into cotton and polypropylene fabrics using a screen-printing technique and it was found that the RL-CuO NPs coated fabrics exhibited significant antimicrobial properties against both Gram-positive and Gram-negative bacteria.	(Haripriya et al., 2024)
Ag@ZnO NPs	The ZnO consisted of individual Ag nanoparticles on the surface of cassia leaf extract as reducing agent and metal surfactant [Co(dpq)_2_(C_12_H_25_NH_2_)_2_] (ClO_4_)_3_ as stabilizer.	Electronic absorption studies showed in the range 400 to 420 nm characteristic sharp absorbance and a single peak suggesting that there is no self-aggregation, whereas the infra-red results show the metallo-surfactant for the stability of the AgNPs due to the presence of metallo surfactant to prevent the agglomeration.	(Nagaraj et al., 2023)
ZnONPs	Zinc oxide nanoparticles (ZnO NPs) were synthesized by a green method using root thickened leaf extract as an effective reducing agent.	It is observed that aqueous extracts of Phlomis leave plant are efficient reducing agents for green synthesis of ZnO NPs in vitro, with no cytotoxic effect on L929 normal cells and a significant impact on the bacteria tested.	(Alyamani et al., 2021)

Differences in preparation methods and types of surfactants used result in variability in the preparation conditions (Singh et al., 2014; Liu, 2008). This study investigates the optimal preparation conditions for zinc oxide nanoparticles (ZnONPs) by exploring the effects of rhamnolipids concentration, reaction temperature, zinc acetate concentration, and calcination temperature through single-factor and orthogonal optimization experiments (Xu et al., 2013). Characterization techniques such as nanoparticle size and Zeta potential analysis, ultraviolet-visible spectrum (Wang et al., 2016), transmission electron microscopy, X-ray diffraction, and Fourier-transform infrared spectroscopy were employed to systematically analyze the products. These analyses ensured that the ZnONPs were successfully modified by rhamnolipids and that high-performance ZnONPs were synthesized.

Harmful fungi like Penicillium citrinum, Aspergillus candidus, Aspergillus flavus, and Fusarium graminearum can contaminate corn during harvest, transportation, and storage (Zhang et al., 2018; Ding et al., 2015). They can enter through contact surfaces, wounds, cracks, and air transmission of spores (Long, 2020). These fungi can thrive in various storage conditions, produce mycotoxins such as aflatoxin, deoxynivalenol, zearalenone, posing a direct threat to human health and life (Wawrzyniak et al., 2018).

The antifungal activity of ZnONPs was evaluated using *Penicillium citrinum*, *Aspergillus candidus*, *Aspergillus flavus*, and *Fusarium graminearum* as test strains. This study represents the first experimental investigation of the key process parameters involved in the modulation of ZnONP morphology, stability, and dispersion by rhamnolipids. The findings present a novel approach for designing nano-antimicrobial agents with enhanced stability and dispersion. Furthermore, this research provides theoretical insights that could facilitate the application of rhamnolipids in nano-based antifungal materials and contribute to the development of new antifungal agents for maize storage.

## Materials and methods

### Materials

The strains *Penicillium citrinum* ATCC1109, *Aspergillus flavus* CA14, *Aspergillus candidus* D20582 and *Fusarium graminearum* ACCC37120 were purchased from shanghai Microbiological Culture Collection Co., Ltd. (Shanghai, China). Zinc acetate and oxalic acid were purchased from Tianjin Yongda Chemical Reagent Co., Ltd. (Tianjin, China). The potato dextrose broth medium (PDB) was purchased from Shanghai Microbial Technology Co., Ltd. (Shanghai, China). Anhydrous ethanol (≥ 99.7%) was purchased from Guoyao Group. Commercially available zinc oxide nanoparticles were purchased from Shanghai McLean Biochemical Technology Co. All other chemicals and reagents employed were commercially available and of analytical grade.

### Preparation of RLs-ZnONPs

**FIGURE 1:**
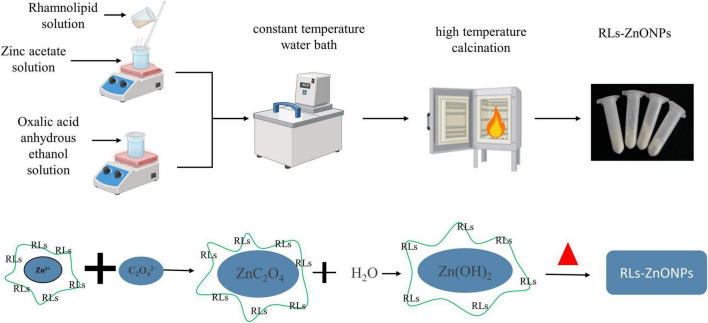
Mechanism of preparation of rhamnolipid-modified zinc oxide nanoparticles.

Refer to the method of Ayeb et al. and make improvements (Ayeb et al., 2021). First, the measured oxalic acid is added to the measured anhydrous ethanol to prepare the oxalic acid anhydrous ethanol solution. The zinc acetate solution was prepared by adding the measured zinc acetate to the distilled water, and the measured rhamnolipids surfactant was added to the solution. Then the solution is placed in a digital constant temperature magnetic stirrer and stirred vigorously to fully dissolve. Then the above prepared zinc acetate solution was added to the oxalic acid anhydrous ethanol solution, and it was placed in a constant temperature water bath for reaction. Following centrifugation, washing, and drying, a white gel was obtained. This gel was subsequently ground and calcined in a muffle furnace to yield a pale yellow powder. The powder was then washed and dried once more to produce RLs-ZnONPs (Figure 1).

### Optimization of preparation process of RLs-ZnONPs

#### Single factor experiment

Define the preparation conditions for zinc oxide nanoparticles through literature review and experimental validation (Piro et al., 2023; Chung et al., 2016; Lee et al., 2009; Kayani et al., 2015). Using ultraviolet-visible spectrum, nanoparticle size analysis, and Zeta potential analyzer results as indicators, investigate the effects of varying concentrations of rhamnolipids (0.6, 0.8, 1.0, 1.2, 1.4 mg/mL), reaction temperatures (50, 60, 70, 80, 90°C), zinc acetate concentrations (0.4, 0.5, 0.6, 0.7, 0.8, 0.9 mol/L), and calcination temperatures (300, 400, 500, 600, 700, 800°C) on the preparation of zinc oxide nanoparticles (ZnONPs). Explore how these factors influence the dispersibility, stability, and particle size of the resultant products.

#### Optimization of orthogonal experiment

On the basis of single factor experiment, L_9_(3^4^) orthogonal experiment was carried out to study the effects of concentration of rhamnolipids, reaction temperature, concentration of zinc acetate and calcination temperature on the dispersion, stability and particle size of the product, so as to determine the optimal process parameters for the preparation of RLs-ZnONPs. Table 2 of test factors and levels was as follows.

**TABLE 2:** Factor and level of orthogonal test.

Level	Factors			
	**A: Concentration of rhamnolipids/(mg/mL)**	**B: reaction temperature/°C**	**C: Concentration of zinc acetate/(mol/L)**	**D: calcination temperature/°C**
1	0.8	50	0.5	400
2	1.0	60	0.7	500
3	1.2	70	0.9	600

### Characterization of RLs-ZnONPs

Dilute the prepared RLs-ZnONPs powder to a concentration of 0.15 g/L. Take an appropriate amount of the diluted sample and transfer it into a sample cell, ensuring uniformity and stability of the sample. Place the sample cell into a nanoparticle size and zeta potential analyzer to measure its nanoparticle size, dispersity, and stability.

Grind the RLs-ZnONPs sample thoroughly and perform X-ray diffraction analysis using a diffractometer under Cu-Kα radiation (wavelength of 1.5406 Å), operated at 40 kV and 40 mA. Scan the sample powder from 20° to 80° (2θ) at a scanning speed of 2°/min to determine the crystallinity and structure of the sample.

Mix the prepared RLs-ZnONPs with potassium bromide (KBr) in a ratio of 1:100 and scan in the wavenumber range of 400-4,000 cm^–1^ for Fourier-transform infrared (FTIR) spectroscopy analysis.

Dissolve an appropriate amount of RLs-ZnONPs powder in a solvent mixture of water and ethanol (1:1 v/v). After ultrasonication, deposit 1-2 drops of the solution onto a carbon-coated copper grid. Allow the solvent to evaporate, then employ transmission electron microscopy (TEM) to observe the morphology and measure the particle size.

### Evaluation of antifungal activity of RLs-ZnONPs

#### Effect of RLs-ZnONPs on the mycelial growth of fungi

RLs-ZnONPs were added to a 250 mL conical flask containing 50 mL PDB, and the final concentrations were 0.0, 1.024, 2.048, 4.096, and 6.144 mg/mL, respectively. The same concentration of rhamnolipids (RLs), commercially available zinc oxide nanoparticles (M-ZnONPs) and unmodified zinc oxide nanoparticles (N-ZnONPs) were used as controls. A total of 1.0 × 10^5^ CFU/mL spore suspensions of *Penicillium citrinum*, *Aspergillus albicans*, *Aspergillus flavus*, *and Fusarium graminearum* were taken and added to PDB medium containing different concentrations of antifungal agents in turn. The culture was oscillated at 28°C and 150 r/min. After 2 days of cultivation, the mycelium was centrifuged and placed in an oven at about 50°C to dry to constant weight.

#### Effect of RLs-ZnONPs on the germination of fungal spores

RLs-ZnONPs were added to a 250 mL conical flask containing 50 mL PDB, and the final concentrations were 0.0, 1.024, 2.048, 4.096, and 6.144 mg/mL, respectively. The same concentration of RLs, M-ZnONPs and N-ZnONPs were used as controls. A total of 1.0 × 10^6^ CFU/mL spore suspensions of *Penicillium citrinum*, *Aspergillus*, *Aspergillus flavus*, *and Fusarium graminearum* were taken and added to PDB medium containing different concentrations of antifungal agents in turn. The culture was oscillated at 28°C and 150 r/min. After 12 h of culture, 20 μL of spore germination liquid was taken in the blood cell counting plate, 100 conidia were observed by optical microscope (40 ×), and the germination rate of fungal conidia was recorded. When the length of the germ tube is half of the width of the conidia, the conidia are considered to germinate and the spore germination is observed (Piermann et al., 2023). The spore germination rate and inhibition rate were calculated:


(1) $$\mathrm{Germination}\mathrm{rate}\left(\%\right)=\frac{A_{2}}{A_{1}}\times 100$$

(2) $$\mathrm{Germination}\mathrm{inhibition}\mathrm{rate}\left(\%\right)=\frac{B_{1}-B_{2}}{B_{1}}\times 100$$

where *A*_1_ is the total number of spores, *A*_2_ is the total number of spore germination, *B*_1_ is the spore germination rate of the control group, *B*_2_ is the spore germination rate of the treatment group.

#### Effect of RLs-ZnONPs on fungal cell membrane

Membrane ergosterol extraction and quantification were done according to some previously published method with some slight modifications (Sharma et al., 2016; Tian et al., 2012). The spore suspension (1.0 mL) of *Penicillium citrinum*, *Aspergillus albicans*, *Aspergillus flavus and Fusarium graminearum* with a concentration of 1.0 × 10^6^ CFU/mL was added to a 250 mL conical flask containing 50 mL PDB and cultured at 28°C and 150 r/min. After 42 h of culture, RLs-ZnONPs were added to the medium to a final concentration of 0.0, 1.024, 2.048, 4.096, and 6.144 mg/mL. After 24 h of shaking culture at 28°C and 150 r/min, the mycelium was filtered and washed three times with PBS buffer, and then dried in the oven to constant weight. The mycelium (0.1 g) was added to 5 mL of 25% KOH-ethanol solution and incubated at 85°C for 4 h. One mL sterile water and 3 mL n-heptane were added. The n-heptane layer was placed by vortex for 2 min, and the ultraviolet-visible spectrum was used to scan at the wavelength of 230 ∼ 300 nm.

#### Effect of RLs-ZnONPs on fungal cell contents

According to the method of Li et al. (2021), The fungal spore solution with a concentration of 1.0 × 10^6^ CFU/mL (2 mL) was inoculated into The PDB liquid medium (100 mL), cultured at 28°C and 200 r/min for 48 h, washed twice with sterile water and resuspended to 40 mL. RLs-ZnONPs were added to make the final mass concentration of 0.0, 0.512, 1.024, 2.048, 4.096 mg/mL, and the culture without RLS-ZnONPs was used as a control. The cells were cultured at 28°C and 200 r/min. At 0, 2, 4, 8, 12, 16, and 24 h, The culture solution (4 mL) was taken to determine the conductivity L/(μS/cm). The absorbance of the supernatant at 260 and 280 nm was measured by spectrophotometer at 0, 2, 4, 6, 8, 10, and 12 h, respectively. The initial conductivity was L_0_/(μS/cm). After 24 h, it was boiled for 10 min and cooled to room temperature. The conductivity L’/(μS/cm) was measured again. The relative conductivity is calculated according to the following formula.


(3) $$\mathrm{Relative}\mathrm{conductivity}\left(\%\right)=\frac{L-L_{0}}{L^{\prime }-L_{0}}\times 100$$

where *L* is the measured conductivity value, *L*_0_ is the initial conductivity value, *L*′ is the conductivity value after boiled.

### Statistical analysis

Data processing and statistical analysis were conducted using The Origin 2022 software (Origin Lab Corporation, United States), and SPSS Statistics 20.0 software (IBM Corp., United States) was used for variance analysis, the difference was significant. All experiments were conducted in triplicate. All data were expressed as mean ± standard deviation (SD). *P* < 0.05 was chosen as the threshold for statistically significant differences.

## Results

### Optimization of preparation process of RLs-ZnONPs

#### Effect of rhamnolipids concentration on the product

**FIGURE 2:**
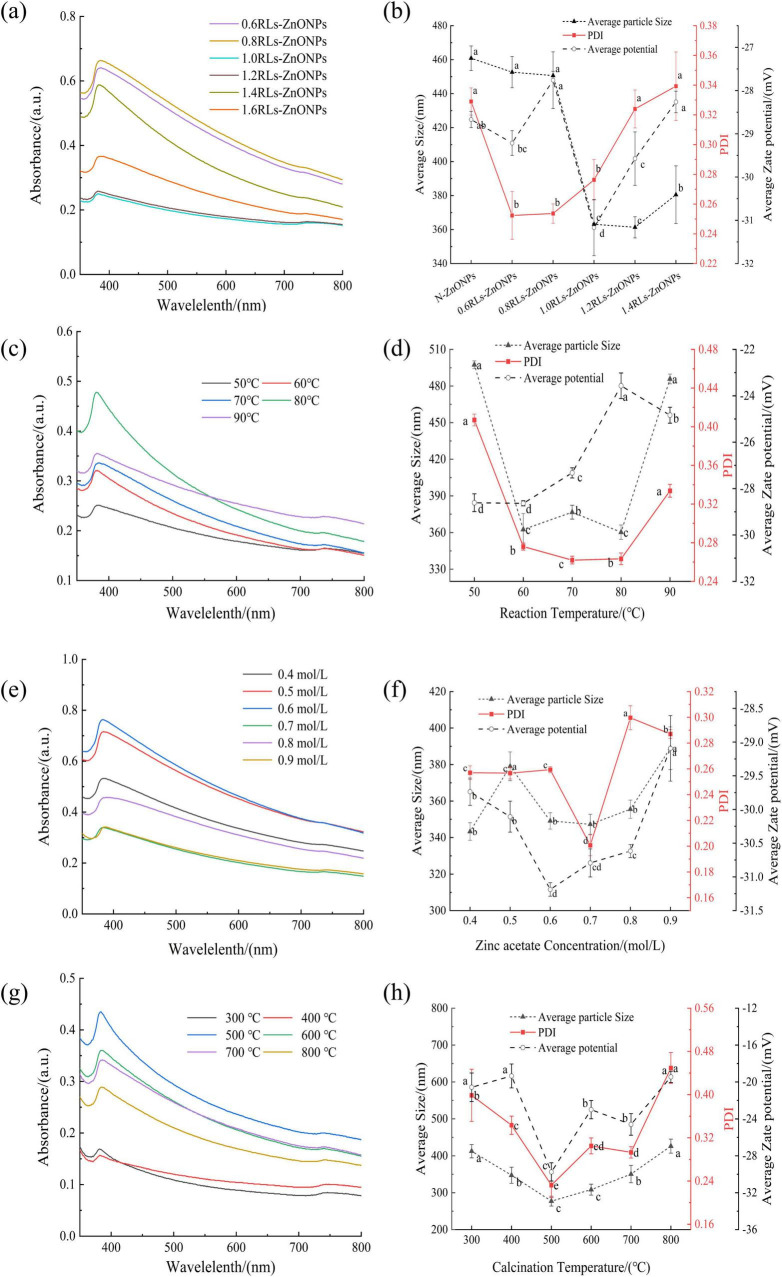
Effect of single factor conditions on the preparation of RLs-ZnONPs. ultraviolet-visible spectrum of RLs-ZnONPs prepared at different RLs concentrations **(a)**, reaction temperatures **(c)**, concentrations of zinc acetate **(e)**, calcination temperatures **(g)**. Nanoparticle size distribution of ZnONPs prepared at different RLs concentration **(b)**, reaction temperatures **(d)**, concentrations of zinc acetate **(f)**, calcination temperatures **(h)**. Different letters in the graph indicate significant differences between the data (*P* < 0.05), as below.

The ZnONPs prepared by adding different concentrations of RLs were scanned by ultraviolet-visible spectrum (Figure 2). With the increase of RLs concentration, the wavelengths corresponding to the maximum absorption peaks of the synthesized products showed a decreasing and then increasing trend (Figure 2a), which were close to the results reported in the literature, and had the characteristic peaks of ZnONPs (Abdelsattar et al., 2023), indicating that the synthesized products were ZnONPs. According to the quantum confinement effect (Gao et al., 2021), when the particle size of the nanoparticles becomes smaller, the energy is converted to the high energy direction, that is, the blue shift phenomenon occurs (Sahraei et al., 2008). In the quantum confinement range, the band gap of the particles increases resulting in the shift of absorption edge to lower wavelength, as the particle size decreases (Singla et al., 2009). The plasma resonance absorption peaks of ZnONPs prepared by adding different concentrations of RLs have a certain degree of blue shift, and the degree of blue shift increases first and then decreases with the increase of RLs concentration, which can indirectly reflect that the particle size of ZnONPs decreases first and then increases.

The nano-particle size of ZnONPs prepared by adding different concentrations of RLs was measured (Figure 2b), with the increase of RLs addition concentration, the average particle size of ZnONPs showed a trend of decreasing first and then increasing. Among them, the average particle size of 1.2 mg/mL reached the minimum value of 361.35 ± 6.26 nm, followed by the average particle size of 1.0 mg/mL RLs-ZnONPs was 363.13 ± 0.52 nm. When the concentration of RLs was 0.6 mg/mL, the Polydispersity Index (PDI) reached the minimum value of 0.252 ± 0.016, indicating that the dispersion of RLs-ZnONPs particles was the best (Bakur et al., 2019), followed by 0.8 and 1.0 mg/mL, and there was no significant difference among the three. When the concentration of RLs was 1.0 mg/mL, the absolute value of the average potential of RLs-ZnONPs increased significantly, indicating that the stability of RLs-ZnONPs solution system could be improved after modification with RLs at a concentration of 1.0 mg/mL (Durval et al., 2021). RLs molecules form a protective layer on the surface of zinc oxide nanoparticles. The hydrophilic group forms a stable hydrophilic interface with the surrounding solution, and the hydrophobic group interacts with the hydrophobic surface of the nano-zinc oxide. This interfacial stability can prevent the aggregation and precipitation of nanoparticles (Cohen et al., 2010). Considering the energy consumption and other issues, the optimal concentration of rhamnolipids was set to 1.0 mg/mL.

#### Effect of reaction temperature on this product

The ultraviolet-visible spectrum of RLs-ZnONPs prepared at different reaction temperatures were scanned (Figure 2c). It can be seen that with the increase of reaction temperature, the wavelengths corresponding to the maximum absorption peaks of the synthesized products showed a decreasing and then increasing trend. Similar to the principle in section 3.1.1, the particle size of RLs-ZnONPs shows a trend of decreasing first and then increasing.

The nano-particle size of RLs-ZnONPs prepared at different reaction temperatures was measured (Figure 2d). It can be seen that when the reaction temperature is 80°C, the particle size of RLs-ZnONPs reaches a minimum of 360.25 ± 5.93 nm. Secondly, when the reaction temperature was 60°C, the average particle size was 362.49 ± 13.04 nm, and there was no significant difference between the two (*P* > 0.05). When the reaction temperature was 70°C, the PDI value of RLs-ZnONPs reached the minimum value of 0.262 ± 0.004. The PDI values corresponding to the temperature of 60 and 80°C were 0.276 ± 0.003 and 0.267 ± 0.003, respectively. When the reaction temperature was 60°C, the absolute value of the average potential of RLs-ZnONPs reached a maximum of 28.64 ± 0.11. Considering the energy consumption and other issues, the optimal reaction temperature is set to 60°C.

#### Effect of Zinc acetate concentration on the product

The RLs-ZnONPs prepared with different zinc acetate concentrations were scanned by ultraviolet-visible spectrum (Figure 2e). With the increase of zinc acetate concentration, the wavelengths corresponding to the maximum absorption peaks of the synthesized products showed a decreasing and then increasing trend. It is preliminarily predicted that when the zinc acetate concentration is about 0.6 mol/L, the prepared RLs-ZnONPs have the smallest particle size.

The nano-particle size of RLs-ZnONPs prepared with different zinc acetate concentrations was determined (Figure 2f). When the concentration of zinc acetate was 0.4 mol/L, the particle size of RLs-ZnONPs reached a minimum of 343.29 ± 4.81 nm. Secondly, when the concentration was 0.7 mol/L, the average particle size of RLs-ZnONPs was 347.27 ± 5.47 nm, and there was no significant difference between the two (*P* > 0.05). In addition, when the concentration of zinc acetate was 0.7 mol/L, the PDI value of RLs-ZnONPs reached the minimum value of 0.201 ± 0.008. When the concentration of zinc acetate was 0.6 mol/L, the absolute value of the average potential of RLs-ZnONPs reached a maximum of 31.19 ± 0.10. When the concentration was 0.7 mol/L, the absolute value of the average potential of RLs-ZnONPs was 30.79 ± 0.21. There was no significant difference between the two (*P* > 0.05). Based on the above test results, the optimal concentration of zinc acetate was considered to be 0.7 mol/L.

#### Effect of calcination temperature on the product

The ultraviolet-visible spectrum of RLs-ZnONPs prepared at different calcination temperatures were scanned (Figure 2g). With the increase of calcination temperature, the wavelengths corresponding to the maximum absorption peaks of the synthesized products showed a decreasing and then increasing trend. It is preliminarily inferred that when the calcination temperature is 400-500°C, the particle size of RLs-ZnONPs is small.

The nano-particle size of RLs-ZnONPs prepared at different calcination temperatures was measured (Figure 2h). When the calcination temperature is 500°C, the particle size of RLs-ZnONPs reaches a minimum of 257.44 ± 5.40 nm. It was observed that while the calcination temperature does not alter the crystal structure of the nanoparticles, it does influence the particle size (Ye et al., 2012). This is attributable to the fact that at lower temperatures, the product undergoes insufficient calcination, whereas at higher temperatures, the polymer network collapses more rapidly, facilitating particle growth as the network no longer restrains it (Rautio et al., 2009).

When the calcination temperature is 500°C, the PDI value of RLs-ZnONPs reaches the minimum value of 0.213 ± 0.038. When the calcination temperature is 500°C, the absolute value of the average potential of RLs-ZnONPs reaches the maximum value of 30.41 ± 0.18, and the optimal calcination temperature is 500°C.

#### Orthogonal experiment

Based on the analysis of the single-factor experimental results, an orthogonal experiment was conducted to determine the optimal preparation conditions for the product. The design factors and their levels are presented in Table 2.

The value of range R indicates the influence of different factors on the index, and the more significant the R, the greater the influence of the factor. The variance analysis showed that the sequence of the degree of the influence factors was as follows: D > B > A > C, that is, the calcination temperature has the greatest influence on the size of the nano-particle size, followed by the reaction temperature, then the rhamnolipidss concentration, and finally the zinc acetate concentration. The optimal process level is A_3_B_2_C_2_D_2_. Taking the polydispersity coefficient as the investigation index, the primary and secondary order affecting the polydispersity coefficient is B > D > A > C, that is, the reaction temperature has the greatest influence on the polydispersity coefficient, followed by the calcination temperature, then the rhamnolipids concentration, and finally the zinc acetate concentration. The optimal process level is A_2_B_2_C_2_D_2_. When the concentration of rhamnolipids is 1.0 mg/mL, the polydispersity coefficient is the smallest, which means that the dispersion of the product is the best, and the particle size of the product at this concentration is relatively small. Considering the problems of energy consumption, the optimal process parameters were set as A_2_B_2_C_2_D_2_, that is, the concentration of rhamnolipids was 1.0 mg/mL, the reaction temperature was 60°C, the concentration of zinc acetate was 0.7 mol/L, and the calcination temperature was 500°C (Table 3).

**TABLE 3:** Orthogonal array design with experimental results.

Number	A	B	C	D	Average particle size/nm	PDI
1	1	1	1	1	504.76 ± 6.75	0.416 ± 0.007
2	1	2	2	2	286.63 ± 6.68	0.188 ± 0.011
3	1	3	3	3	337.01 ± 13.22	0.324 ± 0.010
4	2	1	2	3	360.66 ± 7.73	0.390 ± 0.003
5	2	2	3	1	357.74 ± 7.09	0.292 ± 0.007
6	2	3	1	2	285.53 ± 7.90	0.228 ± 0.002
7	3	1	3	2	321.11 ± 4.26	0.404 ± 0.007
8	3	2	1	3	319.44 ± 3.85	0.317 ± 0.007
9	3	3	2	1	351.05 ± 4.34	0.342 ± 0.002
Average particle size/nm	376.13	395.51	370.24	404.52	D>B>A>C	A_3_B_2_C_2_D_2_
	334.98	321.27	332.78	298.09		
	330.53	324.86	338.62	339.03		
R_1_	45.60	74.24	37.46	106.43		
	0.309	0.403	0.320	0.350		
PDI	0.303	0.266	0.306	0.273	B>D>A>C	A_2_B_2_C_2_D_2_
	0.355	0.298	0.340	0.344		
R_2_	0.052	0.137	0.034	0.077		

### Characterization of RLs-ZnONPs

**FIGURE 3:**
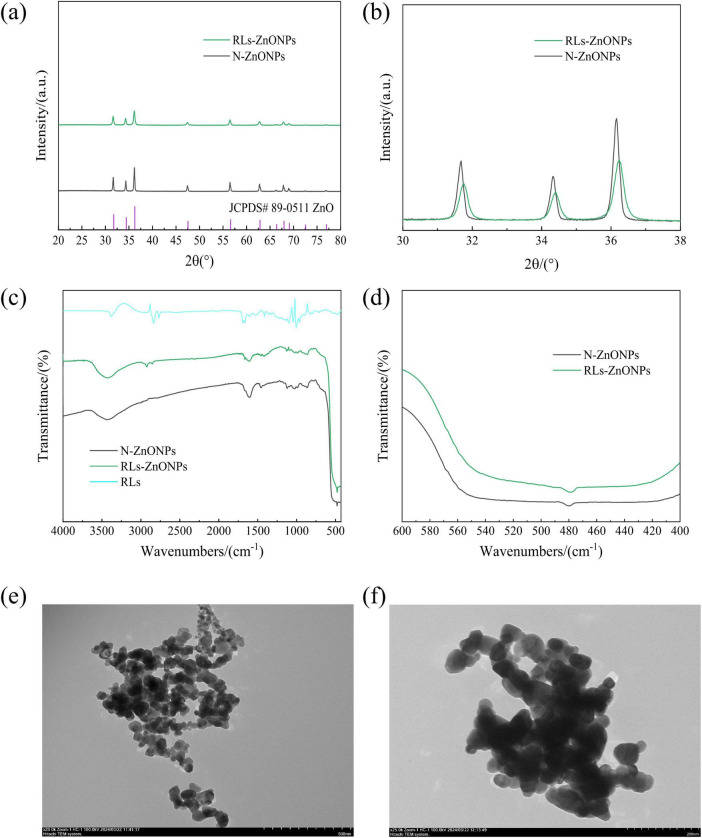
RLs-ZnONPs series characterization diagram. **(a)** Full angle XRD diffraction pattern, **(b)** local angle amplification XRD diffraction pattern, **(c)** Full-wavenumber infrared image, **(d)** local wavenumber amplification infrared diagram, **(e)** transmission electron microscopy of RLs-ZnONPs (20,000 ×), **(f)** transmission electron microscopy of N-ZnONPs (25,000 ×).

The products prepared under optimized conditions were analyzed by X-ray diffraction, Fourier transform infrared spectroscopy and transmission electron microscopy. It was found that the product was consistent with the standard spectrum of JCPDS#89-0511 (Figure 3a), indicating that the prepared ZnONPs were the most stable hexagonal wurtzite structure, the space group was assigned to P63mc, and the lattice constants were a = b = 0.325 nm, c = 0.521 nm, α = β = 90°, γ = 120°. The overall peak of the ZnONPs spectrum is sharp and there is no impurity peak, indicating that the prepared sample has a high purity and a large crystal size (Arciniegas-Grijalba et al., 2019). As the particle size decreases, the XRD diffraction peak of the sample becomes wider and the intensity decreases. It may be that when the grain size decreases to the nanometer scale, the defects in the nanocrystals increase relatively, the lattice spacing changes, and the crystal crystallinity decreases. According to the Scherrer equation, the smaller the grain size, the wider the XRD diffraction peak.

The average particle sizes of RLs-ZnONPs and N-ZnONPs were calculated to be 29.83 and 80.91 nm, respectively. When X-ray is incident on a small crystal, its diffraction lines will become diffuse and broadened (Chai et al., 2007). Compared with the N-ZnONPs prepared without RLs, the diffraction peaks of the samples with RLs moved to the large angle direction, and the interplanar spacing decreased, further indicating that the aggregation between ZnONPs particles decreased (Figure 3b). The particle size of nanocrystals is calculated by the Debye-Scherrer formula (Dutta et al., 2013).


(4) $$D=\frac{0.9\,\lambda }{\beta \,C\,O\,S\,\theta }$$

The products prepared under optimized conditions were analyzed by Fourier transform infrared spectroscopy (Figure 3c). The ZnONPs modified by RLs had an absorption peak at 3421.10 cm^–1^, which was attributed to-OH stretching vibration. The absorption peak at 2854.13 cm^–1^ indicated the stretching vibration of C-H, the absorption peak at 1666.20 cm^–1^ indicated the stretching vibration of ester bond (–COO), the absorption peak at 1417.42 cm^–1^ indicated the bending vibration of methyl C-H, and the absorption peak at 1124.30 cm^–1^ indicated the stretching vibration of C-O, indicating that RLs successfully modified ZnONPs (Singh et al., 2014).

The successful preparation of ZnONPs was confirmed. It was observed that the absorption peak of the product exhibited a red shift phenomenon, indicating a decrease in crystal size and order degree of the nanomaterial structure (Lamiri et al., 2015). This weakening of the crystal field effect led to a narrowing of the energy level interval between the ground state and excited state, resulting in a red shift of the infrared absorption peak. This further supports the conclusion that the particle size of RLs-ZnONPs is smaller than that of N-ZnONPs (Figure 3d).

TEM analysis of the products prepared under optimized conditions showed that the particle size of N-ZnONPs was around 75.0-85.0 nm, whereas the particle size of RLs-ZnONPs was approximately 45-50 nm. The TEM images also indicated that the RLs-ZnONPs exhibited superior dispersion compared to the unmodified N-ZnONPs. Thus, RLs effectively modify ZnONPs, leading to a reduction in average particle size and enhanced dispersion.

### Antifungal activity analysis of RLs-ZnONPs

The optimum preparation conditions of RLs-ZnONPs were determined by single factor and orthogonal optimization experiments, and the antifungal properties of the products prepared under these conditions on the mycelial growth and spore germination rate of *Penicillium citrinum*, *Aspergillus albicans*, *Aspergillus flavus and Fusarium graminearum* were studied.

#### Effect of RLs-ZnONPs on the mycelial growth of fungi

**FIGURE 4:**
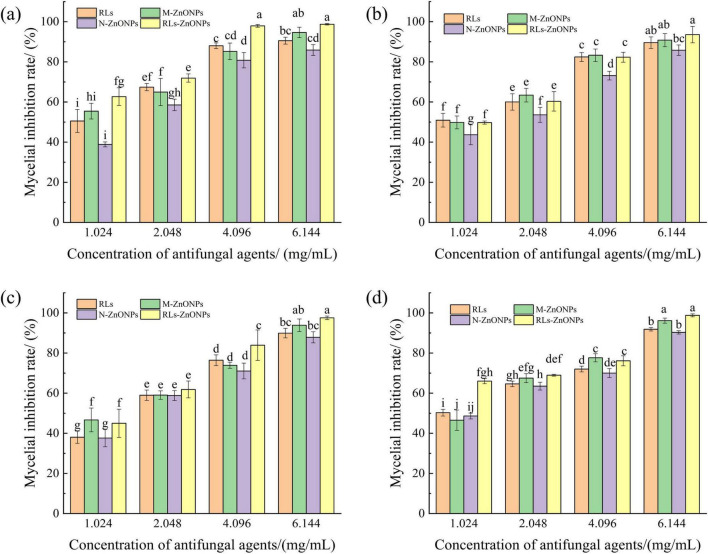
Effect of different concentrations of RLs-ZnONPs on mycelial biomass of fungi. *Penicillium citrinum* **(a)**, *Aspergillus albicans* **(b)**, *Aspergillus flavus* **(c)**, *Fusarium graminearum* **(d)**.

As illustrated in Figure 4, the mycelial growth of the RLs-ZnONPs-treated suspensions of the four fungi showed significant inhibition after 2 days of incubation. In addition, with the increase of RLs-ZnONPs concentration, the mycelial growth of all four fungi was more obviously inhibited. When the concentration of RLs-ZnONPs reached 4.096 mg/mL, the inhibition rate of mycelial biomass of the four fungi reached more than 76.14%. When the concentration of RLs-ZnONPs reached 6.144 mg/mL, the mycelial biomass of the four fungi was inhibited by more than 93.58%, and there was almost no mycelial growth. The antifungal property of RLs-ZnONPs was found to be stronger than that of other control antifungal agents upon comparison. Modification of zinc oxide nanoparticles with rhamnolipids (RLs) showed that RLs-ZnONPs had a significant inhibitory effect on the mycelial growth of the main harmful fungi in maize kernels.

#### Effect of ZnONPs on spore germination of fungi

**FIGURE 5:**
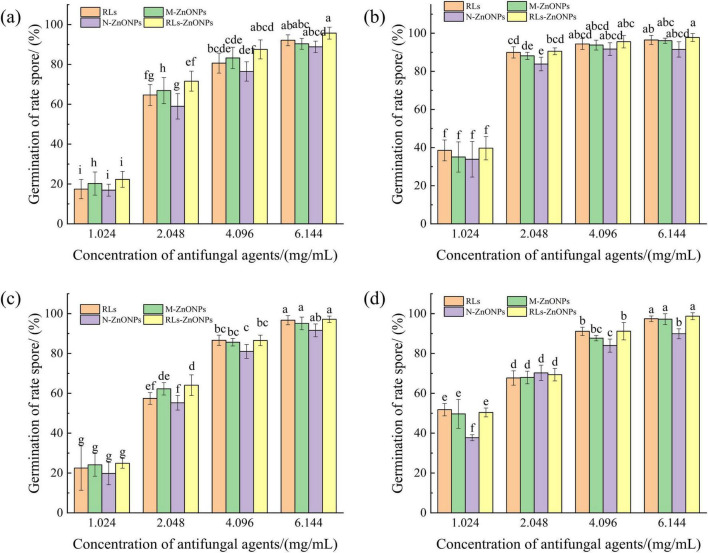
Effect of different concentrations of RLs-ZnONPs on the germination of fungal spores. *Penicillium citrinum* **(a)**, *Aspergillus albicans* **(b)**, *Aspergillus flavus* **(c)**, *Fusarium graminearum* **(d)**.

When the concentration of RLs-ZnONPs reached 4.096 mg/mL, the inhibition rate of spore germination of the four mold species was more than 86.56%. When the concentration of RLs-ZnONPs reached 6.144 mg/mL, the inhibition rate of mycelial biomass of the four molds was more than 95.70%, and the inhibition rate of spore germination of the four molds reached the maximum value when the antifungal agent was RLs-ZnONPs and the concentration was 6.144 mg/mL. There was almost no spore germination (Figure 5). The results showed that the glycolipid-modified products could significantly inhibit the spore germination of the main harmful fungi in grains, thus effectively inhibiting their growth and reproduction. The results showed that the zinc oxide nanoproducts with small particle size, large specific surface area and high surface activity could effectively inhibit fungal spores (Li, 2013).

#### Effect of RLs-ZnONPs on fungal cell membrane

**FIGURE 6:**
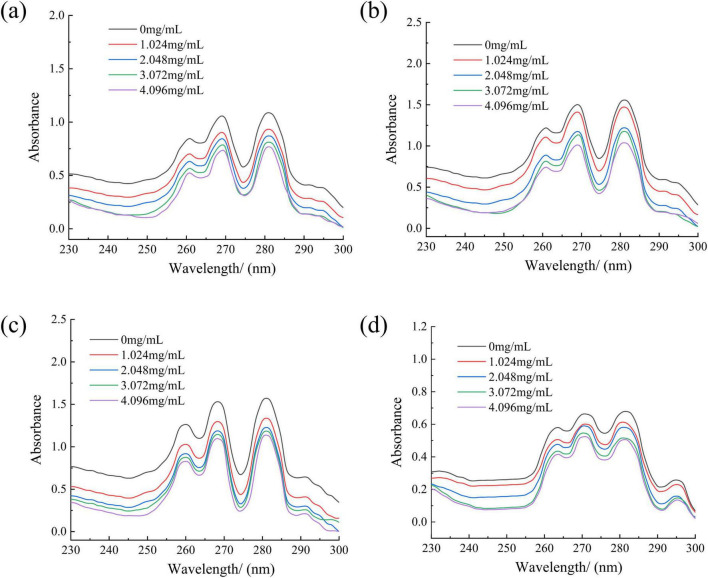
Effect of different concentrations of RLs-ZnONPs on the content of ergosterol in fungi. *Penicillium citrinum* **(a)**, *Aspergillus albicans* **(b)**, *Aspergillus flavus* **(c)**, *Fusarium graminearum* **(d)**.

Ergosterol is an important component of fungal cell membrane, which is essential for maintaining the normal function of cell membrane (Galluzzi et al., 2018). Once the integrity of the cell membrane is destroyed, the cell contents will leak, which may lead to cell damage and death (Li et al., 2013). Fungal sterols have a characteristic absorption spectrum between 240 and 300 nm, which is composed of ergosterol and 24(28)-dehydroergosterol. Both of them have an absorption peak at 281.5 nm. Therefore, the characteristic absorption peak spectrum between 240 and 300 nm and the peak at 280 nm can be used to determine the content of ergosterol (Kocsis et al., 2009). RLs-ZnONPs can reduce the relative content of ergosterol in the cell membrane, and the degree of damage to the cell membrane increases with the increase of the concentration of RLs-ZnONPs (Figure 6).

#### Effect of RLs-ZnONPs on fungal cell contents

**FIGURE 7:**
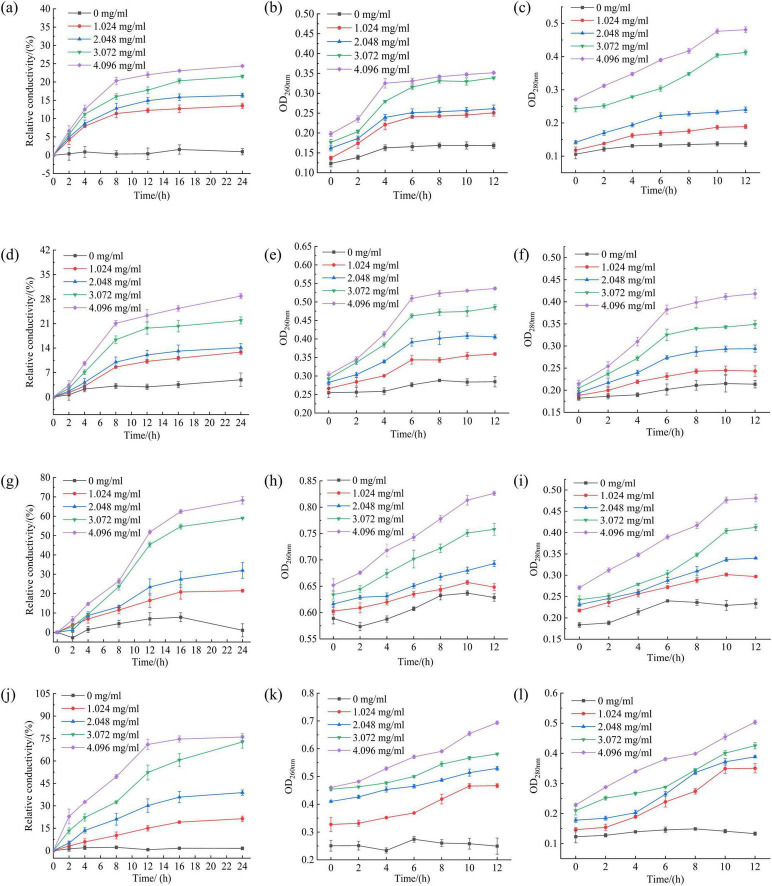
Effect of different concentrations of RLs-ZnONPs on the relative conductivity of *Penicillium citrinum* **(a)**, *Aspergillus albicans* **(d)**, *Aspergillus flavus* **(g)**, *Fusarium graminearum* **(j)**. Effect of different concentrations of RLs-ZnONPs on the OD_260nm_ of *Penicillium citrinum* **(b)**, *Aspergillus albicans* **(e)**, *Aspergillus flavus* **(h)**, *Fusarium graminearum* **(k)**. Effect of different concentrations of RLs-ZnONPs on the OD_280nm_ of *Penicillium citrinum* **(c)**, *Aspergillus albicans* **(f)**, *Aspergillus flavus* **(i)**, *Fusarium graminearum* **(l)**.

The damage of cell membrane led to the leakage of a large number of electrolytes, proteins and nucleic acids (Yang et al., 2024). The conductivity of the control group did not change much, while the relative conductivity of the fungal solution in all experimental groups increased with the prolongation of treatment time and the increase of RLs-ZnONPs concentration. When the concentration of RLs-ZnONPs was 4.096 mg/mL and the treatment time was 24 h, the relative conductivity of *Penicillium citrinum*, *Aspergillus albicans*, *Aspergillus flavus and Fusarium graminearum* suspensions could reach 23.45, 28.87, 68.22, and 76.00%, respectively (Figure 7). The results confirmed that RLs-ZnONPs could increase the permeability of mycelial membrane and cause the leakage of mycelial electrolyte.

Protein and nucleic acid are important macromolecular substances in cells, which play a decisive role in the growth and reproduction of bacteria (Yao et al., 2014). The release of nucleic acid and protein in cell contents was analyzed by measuring the optical density values (OD_260nm_ and OD_280nm_) at 260 nm and 280 nm wavelengths (Gao et al., 2023). The OD_260nm_ and OD_280nm_ of the control group showed a low level, while the OD_26nm_ and OD_280nm_ of the fungal suspension treated with RLs-ZnONPs increased significantly, and increased with the increase of RLs-ZnONPs concentration and the prolongation of action time (Figure 7), indicating that RLs-ZnONPs had a significant destructive effect on the permeability and integrity of the cell membrane, resulting in a large amount of leakage of nucleic acids and proteins, causing dysfunction and affecting cell growth.

## Discussion

The UV–vis absorption of the hexagonal ZnS nanospheres prepared with the assistance of alginic acid shows a strong and sharp excitonic peak at 317 nm, compared with that of the bulk wurtzite ZnS, which is blue shifted by about 0.11 eV. Since the obtained ZnS nanosphere is composed of small nanoparticles with an average size of about 4–5 nm, which is comparable with the Bohr diameter of bulk ZnS (5 nm). This result bears certain similarities to the findings of the present study (Hou and Gao, 2011). In a study by Barhoum et al., which employed the sol-gel method to prepare ZnO nanoparticles loaded onto a porous silica matrix, it was found that the temperature of the reaction medium is a critical factor influencing the formation of ZnO and SiO_2_, crystal growth, crystallization processes, and phase transitions. Specifically, adjusting the reaction temperature can significantly impact the structural characteristics and performance of the nanomaterials, a finding that bears notable similarities to the results of the present study (Barhoum et al., 2017). In the sol-gel preparation of nanostructured zinc oxide, the concentration of zinc acetate plays a crucial role in determining the properties of the product. Varying concentrations of zinc acetate not only influence the grain size and morphology of the zinc oxide but also regulate its structural characteristics by introducing crystal defects. The presence of these defects can significantly alter the physicochemical properties of the zinc oxide (Bouderbala et al., 2024). Micrographs and particle size analysis reveal that particle sizes are predominantly influenced by the calcination temperature, where a plate-like morphology at lower temperatures gradually transformed into complete nanorods at 700°C, with the reduced agglomeration and wide particle distribution (Sangeetha et al., 2019).

Existing studies have shown that metal oxides exhibit excellent antimicrobial activity (Correa et al., 2020). Among these, nanoparticles of gold, silver, and other metal oxides have been proven to possess antimicrobial properties (Varghese et al., 2024; Moradialvand et al., 2024; Holubnycha et al., 2024; Caselli et al., 2024). Suganya et al. developed a potent antifungal nanocomposite with NiO NPs against the Aspergillus niger strain. The authors attributed the excellent antifungal properties to the physical process used to internalize the powdered nanomaterial in the fungi cells and also to the chemical process that involved ROS generation (Suganya et al., 2018). Zinc oxide nanoparticles also demonstrate antimicrobial effects and, in comparison, offer the advantage of reduced production costs. It was found that the mechanism of antifungal activity of ZnO through physiological changes, the probable mechanism is that ZnO acts directly on the mycelium, generating oxidative stress and disrupting the intracellular physiological equilibrium, and the antifungal mechanism is attributed to the oxidative stress and changes in membrane function (Zhang et al., 2019). Most current research on the antimicrobial properties of zinc oxide nanoparticles has been primarily focused on foodborne pathogens such as *Escherichia coli* and *Staphylococcus aureus* (Varghese et al., 2024; Caron et al., 2024; Park et al., 2024), with limited studies on their antifungal activity. Additionally, the antimicrobial mechanisms of zinc oxide nanoparticles remain to be fully elucidated. This study demonstrates that rhamnolipids-modified zinc oxide nanoparticles exert antifungal effects by inhibiting fungal cell membrane synthesis, disrupting membrane integrity, and inducing massive leakage of intracellular electrolytes, nucleic acids, and protein content, thereby achieving potent antimicrobial activity.

## Conclusion

The optimal preparation conditions of RLs modified ZnONPs were determined by single factor and orthogonal optimization experiments to obtain RLs-ZnONPs with the smallest particle size, the best dispersibility and the best stability. The results showed that when the concentration of RLs, reaction temperature, zinc acetate concentration and calcination temperature were 1.0 mg/mL, 60°C, 0.7 mol/L, and 500°C, respectively, the average particle size of RLs-ZnONPs was about 45-50 nm, the minimum PDI value was 0.213 ± 0.038, and the maximum absolute value of average potential was 30.41 ± 0.18. The antifungal properties of RLs-ZnONPs were evaluated. The results showed that when the concentration was 4.096 mg/mL, the inhibition rate of mycelial biomass of the four fungi reached more than 76.14%. When the concentration of RLs-ZnONPs reached 4.096 mg/mL, the inhibition rate of spore germination of the four molds reached more than 86.56%, indicating that RLs-ZnONPs had a good antifungal effect on fungi. By measuring the content, conductivity, OD_260nm_ and OD_280nm_ of ergosterol in fungi, the results showed that RLs-ZnONPs could inhibit the synthesis of ergosterol in four fungi, increase the conductivity of mycelium suspension, and increase the OD_260nm_ and OD_280nm_ values, indicating that RLs-ZnONPs could destroy the cell membrane of the bacteria, leak the nucleic acid and protein content of the bacteria, so as to achieve the bacteriostatic effect.

This study initially addresses the challenges of agglomeration and instability in the preparation of nano-zinc oxide, effectively regulating its particle size and offering a reference for the use of glycolipids in the green synthesis of nano-antifungal materials. Additionally, it provides insights into the application of nano-zinc oxide for bacteriostatic purposes and preliminarily explores its antifungal mechanisms. In the field of agriculture, uniform spraying of ZnONPs solution on stored corn kernels can effectively prevent the growth of harmful fungi on its surface, which helps to promote the application of ZnONPs in grain or feed storage, and coating seeds with ZnONPs can prevent the fungi in the soil from infecting seedlings (Azeez and Barzinjy, 2020). In the industrial field, ZnONPs are incorporated into paints, plastics or textiles for antifungal preservation of medical devices, food packaging or construction materials.

ZnONPs have significant potential for antifungal applications in agriculture and industry, and their high efficiency, environmental compatibility and versatility offer new directions for replacing traditional chemicals. However, further research is required to thoroughly investigate its antifungal properties and elucidate the mechanisms underlying its antifungal activity.

## Data Availability

The original contributions presented in the study are included in the article/supplementary material, further inquiries can be directed to the corresponding author.
